# Media literacies, platform imaginaries and mental health influencers: How young people respond to mental health content on TikTok

**DOI:** 10.1177/20552076261479043

**Published:** 2026-08-21

**Authors:** Alex Christiansen, Ruth Page, Shioma-Lei Craythorne, Michael Larkin, Paul Crawford, Evangelina Asiedu-Addo, Emma Garavini

**Affiliations:** 1 1724 School of Arts and Law, Department of Linguistics and Communication, University of Birmingham, Birmingham, UK; 2 1722 School of Psychology, Health and Clinical Sciences, Institute of Health and Neurodevelopment, Aston University, Birmingham, UK; 3 6123 School of Health Sciences, Division of Nursing, University of Nottingham, Nottingham, UK; 4 596019 The McPin Foundation, London, UK

**Keywords:** media literacy, mental health, influencers, thematic analysis, social media analysis, tiktok

## Abstract

**Objective:**

Social media is increasingly used as a source of mental health information for young people, with influencers playing a key role in public health communication. However, little is known about how young people engage with influencer mental health content and the media literacy skills they use to evaluate it. The objective of this study is to explore and evaluate the self-reported media literacy skills and beliefs that young people use when they encounter mental health content on TikTok.

**Methods:**

The study uses qualitative, thematic analysis to provide an empirical account of how young people interpret mental health content from influencers on TikTok. Research was conducted via eighteen group (n = 73 participants) and individual interviews (n = 13) with eighty-six participants in the United Kingdom. The analysis focused on self-reported media literacy skills and strategies for navigating influencer-led mental health content.

**Results:**

The study identifies three contextual challenges in young people’s responses to mental health content on TikTok: (I) reliability and relatability act as distinct but complementary criteria used to assess influencers and their mental health content; (II) platform beliefs influence willingness to engage with mental health content; and (III) algorithmically driven platform design hinders critical thinking about mental health content.

**Conclusions:**

These findings highlight the sophistication of young people’s media literacy skills alongside the significant, contextual barriers, which hinder the use of social media as a channel for mental health support from influencers. Our results suggest that public health campaigns may need to take a more platform-sensitive approach to online communication, acknowledging the ways platform-driven beliefs and design shape how and why young people engage with mental health content.

## 1. Introduction

The gap between the number of people living with mental health conditions and those accessing professional services is an urgent social and economic challenge. In 2025, the World Health Organization^1^ reported that although there are over 1 billion people globally who experience mental health disorders, governmental spending on support for services in this sector has stagnated. In the United Kingdom, it is estimated that one in five young people had a probable mental disorder in 2023,^2^ while people are eight times more likely to wait over 18 months for mental health treatment, compared to physical health treatment.^3^ Social media is increasingly used as an alternative source of mental health information^4,5^ and taken up as a form of ‘DIY support’ especially by young people.^6^

Social media influencers are known to impact the lives of adolescents^7^ as aspirational role models^8^ who impact attitudes through their advertising.^9^ Distinct from celebrities, influencers are characterised by their relatable personalities combined with high visibility.^10^ Within health communication, relatability is recognised as desirable quality needed for mental health support within peer networks^11^ and used as an interpersonal strategy by mental health professionals as part of therapeutic practice.^12^ As such, they may be one route to reaching young people with online public health communication and support for mental health challenges. Early studies explored the possibility of collaborating with influencers as opinion leaders who raised awareness of health topics such as vaccines,^13^ or as sources of health education.^14^ Increasingly, the engaging styles used by influencers are promoted as the new paradigm for health communication,^15^ seen in global initiatives, such as the World Health Organization’s Fides network.^16^

In their exploratory research, Pretorious et al.^17^ analysed the content of a small sample of mental health professionals (n = 28) who as influencers posted on TikTok and Instagram, finding that a quarter of the videos contained educational materials with the potential to support mental health literacy, a key skill for young people in transition to adulthood.^18^ However, influencers who share information and advice about mental health on social media also include wellness figures^19^ and lay persons who document personal feelings and experiences of mental health.^20^ These lay figures are reportedly superseding the use of search engines like Google as sources of medical help.^21^ Scoping reviews found that across this wider range of influencers, many lacked health expertise and provided inaccurate advice,^22,23^ and there are emerging concerns over deceptive advertising of mental health therapy.^24^ There are promising studies that show that, with training, influencers can boost evidence-based mental health content on sites like TikTok.^25^ Participant-based perspectives on influencer content are only beginning to emerge, such as Adeane and Stasiak’s study of young people’s attitudes to influencers as sources of mental health content in New Zealand.^26^ We now need platform-specific, in-depth exploration of the skills that young people from other national contexts use to navigate mental health content from influencers. Qualitative methods are best suited to this endeavour, as they enable researchers to uncover the interpretative processes that underpin responses and decision making, offering a more nuanced, fine-grained understanding of how people navigate, evaluate and make meaning from mental health content online.^27^

Given that influencers communicate via various social media sites and platforms, media literacy plays a key role in this picture.^28^ Media literacy is defined as ‘the ability to use, understand and create media and communications across multiple formats and services’.^29^ Media literacy is multifaceted. Researchers have explored its different aspects, including knowledge and use of functional skills,^30^ beliefs and attitudes towards technology,^31^ and understanding of how data are used.^32^ Critical evaluation skills have been studied in relation to practices of consumption,^27,33^ curation^34^ and disengagement from digital content.^35,36^ More specialized forms of media literacy have been explored from these perspectives, such as multimodal literacy, that is, the skills and competencies needed to engage critically with meaning across different modes of communication (visual, verbal and so on)^37^ information literacy, that is, the ability to navigate, locate and evaluate information in digital environments^38^ and algorithmic literacy, that is, awareness of, attitudes and behaviour towards algorithmic media.^32^

We take a comprehensive approach to media literacy that brings together the ways skills, practices and beliefs are negotiated by young people in relation to different sites and platforms. This builds on concepts from media studies including platform imaginaries,^39^ or ‘what users *think* a platform is and does’^40^ that shape the purposes for which sites are used and how platform affordances are perceived and taken up. We study the latter through affordance theory. In their recent systematic review of the literature Ronzhyn et al.^41^ argue that from a relational approach, affordances go beyond the specific functions of the technology and should recognize the agency involved in individual’s uses of technology. We follow their definition of social media affordances as ‘the perceived actual or imagined properties of social media, emerging through the relation of technological, social, and contextual, that enable and constrain specific uses of the platform’.^41^ As such, we take the overarching term ‘media literacy’ to encompass how people understand the affordances of particular sites and the functional skills they use when engaging with them; people’s beliefs and dispositions about platforms; the types of critical thinking that people bring to bear when engaging with different platforms for different purposes.

We focus on TikTok as the most popular social media app used by children and young people aged 4-18 in the United Kingdom.^42^ The affordances of TikTok have begun to be explored in relation to healthcare information^43^ and information literacies.^44^ TikTok is a video-centric app, which includes options for its members to engage with content through searching, viewing, curating, commenting, and reposting practices. The app is distinctive for its algorithmically driven recommendations, which promote content to the viewer through the personalized feeds that function as the app’s default view: the ‘for you page’. TikTok has also attracted interest from scholars who have pointed to the risks of health misinformation circulating through viral sharing on the platform,^45^ including the potential for misinformation to prompt help-seeking from non-evidence based treatments for neurodevelopmental conditions such as ADHD.^46^ A recent meta-analysis^47^ found that media literacy improved wellbeing. However, misinformation about mental health remains relatively under scrutinized within media literacy scholarship.^48^ Influencers such as wellness practitioners and recovery accounts also use personal stories as a persuasive strategy associated with relatability,^49^ which may be misleading but fall outside the boundaries of misinformation.^48^ We urgently need to better understand the media literacy skills that young people use when navigating mental health content from a range of influencers whose content proliferates on this site.

In response to recent calls for media literacy to support people accessing health content from influencers,^50^ the objective of our study is to explore how young people evaluate the mental health content they encounter from influencers on TikTok. We address the following research questions:

RQ1: How do young people’s media literacies inform their response to content from mental health influencers on TikTok?
RQ2: What role do young people’s beliefs about platforms play in shaping how they might engage with mental health content from influencers?

## 2. Methods

### 2.1. Study design

The exploratory, qualitative design centred on young people’s perspectives and self-reported practices as they responded to mental health content shared by social media influencers. The study took place in the United Kingdom, between October 2024 and May 2026. We took a social constructionist approach, recognising that our perspectives shaped how we understood the data. Across the team, the interviewers varied in terms of age and gender. We also come to the topic from different disciplinary and methodological backgrounds. AC and RP are discourse analysts; SLC and ML are psychologists; PC works in public health communication, EG and EA are peer researchers and youth involvement officers. None of us are young enough to have shared the social media environment that our participants now inhabit. We worked closely with the project’s Young People’s Advisory Group (YPAG), to help us better understand this fast-changing online context and took an open stance towards the interviews and analysis.

The YPAG included twelve young people aged between 16 and 25 with lived experience of mental health challenges, issues, and experience of using and engaging with social media and social media influencers (e.g. TikTok & Instagram). The YPAG was recruited and facilitated by the McPin Foundation, a mental health charity that specialize in championing and working with lived experience of mental health challenges. The YPAG met four times, remotely, during the study and contributed to the research design from the outset. They reviewed the language in the recruitment letters sent to educational institutions for the group interviews and adverts used for the individual interviews. All members of the YPAG short-listed the videos shown as discussion prompts in the topic guides (see Appendix A). Four members gave detailed feedback on the activities, structure and wording used in the group and individual interviews. For example, they suggested preparatory activities such as icebreakers, definitions of the term ‘mental health influencer’ and debriefing opportunities at the end of each session. The initial analysis of the group and individual interviews were shared with in a project meeting with the whole YPAG in May 2025, where the YPAG members drew on their own experiences and views of social media content and literacy skills to further support the themes developed in the coding.

### 2.2. Sampling strategy and recruitment

We aimed to include a diverse range of participants aged between 16-25 years, who as young and emerging adults experience significant life transitions,^51^ are likely to benefit from mental health support via social media^52^ and were the largest group of TikTok users at the time of our study.^53^ To ensure that we gathered perspectives from participants enrolled in full time education as well as those who were not, and to allow multiple formats for participation, we offered group and individual interviews using the following recruitment strategies.

We chose to engage and recruit the young people via schools and colleges in the Midlands, England. We sampled schools purposively based on their catchment areas and intake profiles, writing to the head teachers of fourteen educational organizations, including a cross-section of selective/non-selective schools, faith-based schools, FE colleges, and charities to reach those outside formal education. Twelve organizations agreed to take part. Young people were sampled opportunistically within those schools via teachers and senior leaders. We offered both group and individual interviews within the schools and colleges. Only group interviews were taken up. No individual interviews took place in schools and colleges. We also offered individual interviews for participants recruited via the McPin Foundation Young People’s Network mailing list. Eligible participants were English-speaking, aged 16–25, with an interest in social media. We recognise this means the sample is likely to be biased by self-selection, which may result in social desirability influencing some of the responses that were given. All participants were English-speaking and received a £25 Amazon e-voucher; notably, prior experience with mental health challenges was not a requirement for either participant group.

### 2.3. Sample composition

Eighteen group interviews with 73 participants were conducted in participating schools and colleges by three of the research team (AC, RP, SLC), including male and female members, trained in qualitative methods and PPI research principles. The group interviews took place in participating schools and colleges between November 2024 and February 2025. In four group interviews (Groups 1, 2, 9 and 10) teachers were present. Saturation was reached after sixteen groups, after which no new variations in the participants’ insights about influencers were observed. Two further groups were conducted to confirm this. Individual interviewees (n = 13) were recruited in December 2024 and conducted as online interviews via Microsoft Teams by SLC, with both cameras active throughout. Data saturation was reached after twelve interviews, with one further interview conducted to confirm this. No repeat interviews were carried out. See Table 1 for sample characteristics.

**Table 1.** Demographic data across interview types.

Demographic category	Group interviews (n = 73)	Individual interviews (n = 13)
**Gender Identity**		
Female	53.4% (n = 39)	61.5% (n = 8)
Male	39.7% (n = 29)	38.5% (n = 5)
Other	1.4% (n = 1)	0%
Prefer not to state	5.5% (n = 4)	0%
**Ethnicity**		
Asian	42%	23.1% (n = 3)
Black	15%	46.2% (n = 6)
White British	24%	23.1% (n = 3)
Mixed/Other	12%	7.7% (n = 1)
White European/Other	7%	0%
**Education (Highest Level)**		
Degree or higher	1.6% (n = 2)	38.5% (n = 5)
A-levels/Level 3	8.6% (n = 11)	15.4% (n = 2)
GCSE/Level 1–2	75.8% (n = 97)*	7.7% (n = 1)
Mixed/Other Pathways	10.2% (n = 13)*	38.5% (n = 5)
No Qualifications	0.8% (n = 1)	0%
**Additional Needs**		
Disability	5% (n = 4)	15% (n = 2)
Learning Difficulties	7% (n = 5)	15% (n = 2)

The average group interview had 4 participants aged between 16–18, with a female majority (53.4%) across groups, accounting for variation between schools. The sample was ethnically diverse, with Asian (42%), White British (24%), and Black (15%) participants most represented. The majority of group interview participants reported GCSE or equivalent Level 1–2 qualifications as their highest level of education (75.8%), consistent with the 16–18 age range. Audio recordings of the interviews averaged 46 minutes, not counting the time participants spent filling out pre-group interview surveys, learning about the basis of the research, and engaging in ice-breaker activities. The individual interview participants were aged 18-25 (mean age 21.6, SD = 2.6) and the audio recordings were slightly shorter at an average of 40 minutes each. This sample was similarly female-majority (61.5%) but more educationally mixed, with degree-level qualifications (38.5%) and mixed or other pathways (38.5%) most common.

### 2.4. Data collection

The semi-structured topic guide used in both group and individual interviews consisted of three stages. First, we focused on past experiences of engaging with influencer content and familiarity with mental health influencers. In the second section, three videos from influencers sharing content about anxiety were used as discussion prompts. We used the three influencer categories based on our previous analysis of TikTok videos^49^: mental health professionals, wellness practitioners and lay persons. From these categories, three representative videos about anxiety were shown to serve as discussion prompts: a psychiatrist detailing clinical signs of social anxiety, a wellness influencer discussing nutrition-based treatments, and a lay person’s 'Day in the Life’ recovery story. The interviewers then asked open-ended questions about the perceived value of the videos, using prompts focused on information, attitudes and support for mental health. Participants were then asked to reflect on how they would engage with the videos and their reasons for this. In the third section of the interview, participants were invited to share the priorities they felt should be addressed by policy makers concerned with public health communication from influencers. The topic guide was tested with a group of three participants ahead of its use in the data collection. Table 2.

**Table 2.** Indicative questions and prompts from semi-structured topic guide.

Question	Possible prompts
Could you tell us about some influencers you have heard about or seen?	• How did you come across them? • Why do you feel that way about them?
Please rank order videos to indicate how useful is for providing information about mental health.	• What did you find helpful (or not) about each video?
For each video: How would you interact this video if you saw it online?	• Would you scroll past it? • Would you save it to watch later? • Would you send it to a friend? Or anything else? How did you feel when you watched this?
What would you most like policy makers to know about how mental health communication from influencers is shared in social media?	• What works well? • What most needs to change?

Following interviews, participants were offered to self-report personal demographics (gender identity, ethnicity, belief system, disability, learning difficulties) alongside their own and their parent, guardian or carer’s qualifications and occupations. Each researcher kept observation notes following each interview, recording initial points of interest.

### 2.5. Ethical issues

Approval was obtained from the Humanities and Social Sciences Ethical Review Committee at the University of Birmingham (ERN_23-0961P) and governance approval granted from the Research Integrity Office at Aston University (ERN_23-0961AP2) to perform group interviews and individual interviews with young people aged 16-25 (1 November, 2024). Each participant provided written consent to participate. With advice from our project partners at the McPin Foundation, we developed and followed a distress protocol to ensure that we responded promptly and appropriately to any evidence of distress or disclosures of risk. There were no significant incidents during data collection that required escalation or referral to third parties.

### 2.6. Data analysis

The group interview recordings were transcribed by a third party under a confidentiality agreement. The individual interviews were recorded via Teams. All data were anonymized and were checked by the researchers for accuracy. Transcripts were stored securely on the University of Birmingham’s institutional One Drive platform, a secure, GDPR-compliant cloud storage system. Access was restricted to authorised members of the research team (AC, RP, SLC). Because the data were anonymized at the point of transcription in line with GDPR, it was not possible return the transcriptions of the group interviews for checking by participants. For consistency, this principle was also applied to the individual interviews.

We employed a six-step process designed to facilitate Reflexive Thematic Analysis,^54^ whilst maximizing the opportunities to check the plausibility and coherence of our analysis as we developed it. An overview of the process can be seen in Table 3. During the data familiarization and line-by-line coding, broad similarity between the group and individual interviews was observed. The full coding using the same code book was then applied to both group and individual interview transcripts.

**Table 3.** Overview of six-phase coding process.

Step	Activity	Data	Authors involved
1	Data familiarization	Authors read the entire dataset	AC, SLC, RP
2	Line-by-line coding of semantic and latent codes, inductively oriented from the data	8 longest group interviews, 13 individual interviews	AC, RP (group interviews), SLC (individual interviews)
3	Initial themes generated by the analysis team in two meetings	Coding from group interviews	AC, SLC, ML, RP
4	Coding expanded (total of 155 codes) to develop the six themes	Entire dataset coded in NVivo 14.23.2	RP
5	Themes reviewed and refined to focus on three media literacy themes and 50 codes.	Entire dataset	AC, RP, ML, SLC
6	Writing up the analysis of the media literacy codes with feedback from the whole authorship team	Entire dataset	AC, RP, ML, EA, EG, SLC, PC

## 3. Results

The analysis produced three intersecting themes: (T1) Reliability and relatability are criteria for assessing mental health content, (T2) Platform beliefs position TikTok as a site that is fun but fake, (T3) Platform design is tricky. Conceptually, these themes reflect the different focus for the self-reported media literacy skills used by young people to respond to mental health content from influencers. Theme 1 focuses on content, while themes 2 and 3 are oriented to contextual concerns, as indicated in Figure 1.

**Figure 1.**
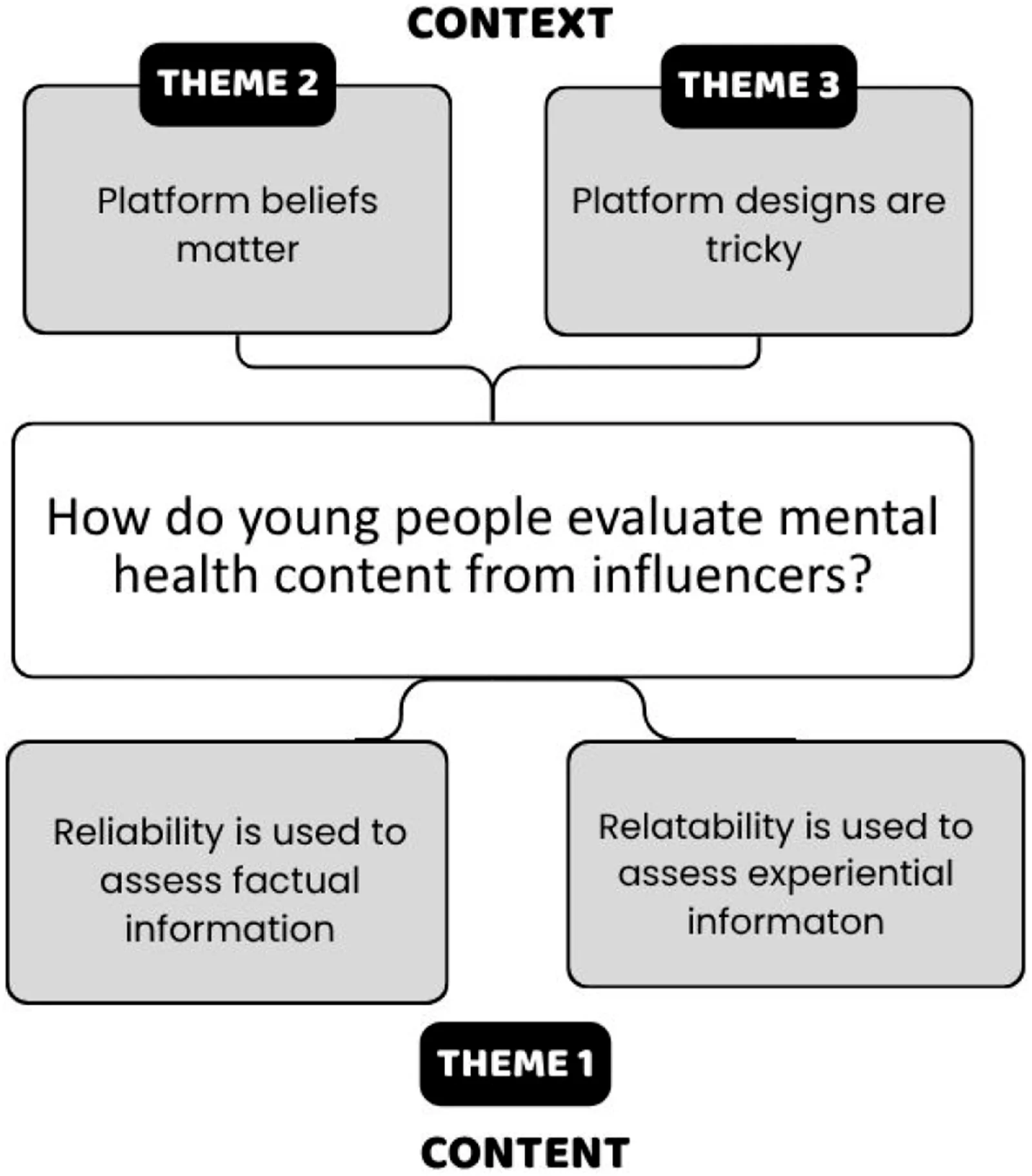
Theme map.

Quantitatively, the coding was distributed almost equally between the media literacy skills focused on content, with 148 references coded to Theme 1 and 146 references coded to the combined contextual themes, with Theme 2, exploring the role of platform beliefs accounting for 83 references and Theme 3, platform design, accounting for 3 references. The quantitative distribution of these codes is visualised in Figure 2. Each of the themes is explored in more detail below.

**Figure 2.**
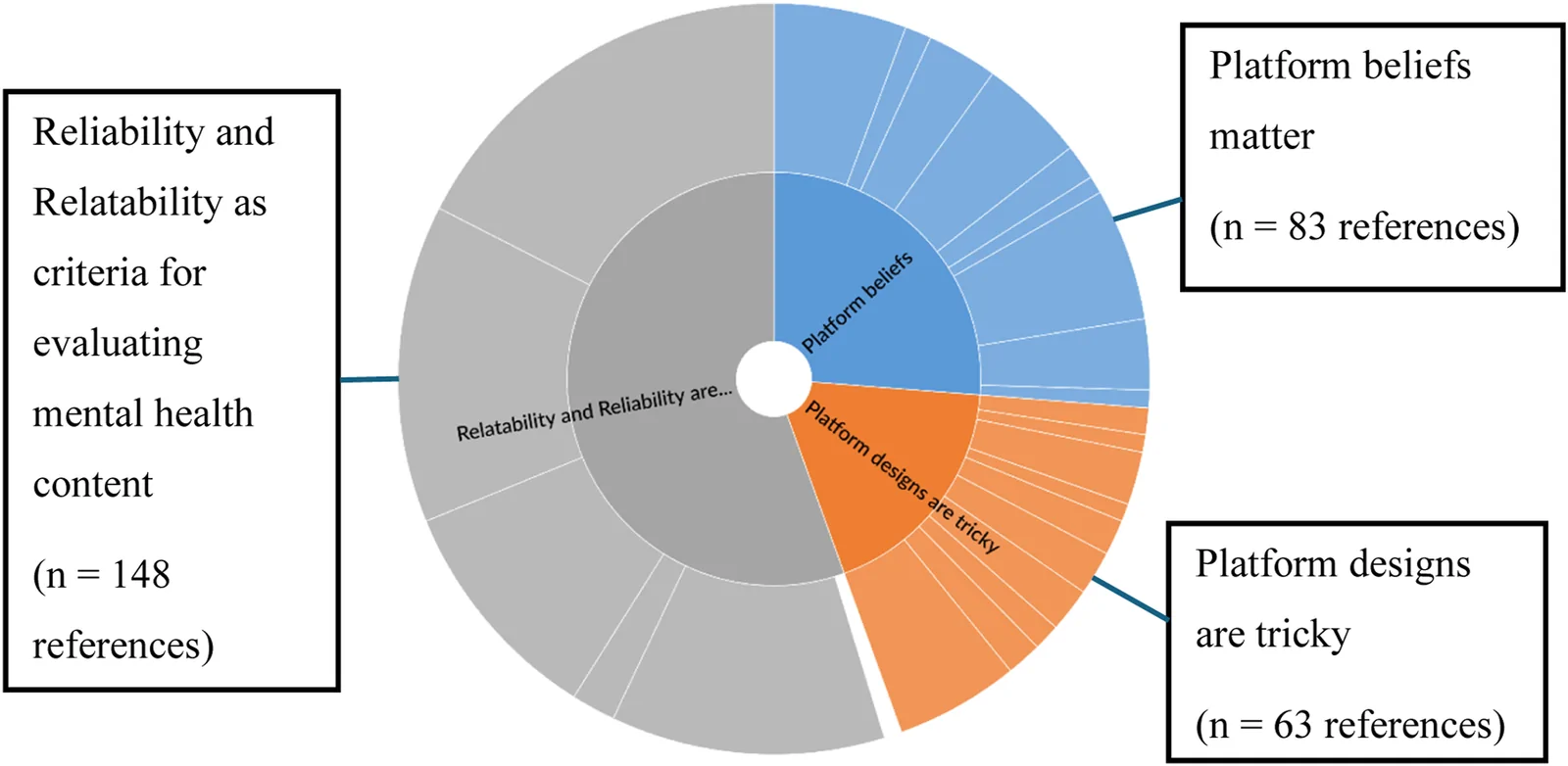
Sunburst chart produced in NVivo 14.23.2 showing the quantity of references for the three over-arching media literacy themes.

### 3.1. Theme 1. Reliability and relatability are criteria for assessing mental health content

In their critical evaluation of mental health content, the young people in our study drew on reliability and relatability as distinct, separable criteria applied to different kinds of content. Reliability was used to evaluate ‘factual’ (Group Interview 1) information about mental health issues and treatments, assessed in relation to evidence such as ‘proof’ (Group Interview 15) and ‘research’ (Group Interview 5). Young people deemed inaccurate, unsubstantiated or misleading content risky, a source of ‘suffering’ and ‘a really big thing’ (Group Interview 16). The reliability of factual mental health content was associated with the authority of the content creator. The professional role of the influencer, inferred through ‘titles’ and ‘qualifications’ was taken as a sign of valued credibility.

I feel like having a doctor online giving you information is really useful because they’ve got qualifications and experience (Group Interview 3).

However, for others, simply stating credentials was not enough to know whether a content creator was ‘an actual doctor or not’ (Group Interview 6). Claims to be a professional also needed verification. As one participant in Group Interview 18 explained, ‘He slapped the word doctor in front of his name, but like there’s no proof.’

Nonetheless, factual information from health professionals was considered widely applicable, with content aimed ‘at everyone’ (Group Interview 8). Doctors were assumed to have breadth of professional experience, which was valued as providing a more objective ‘outsider perspective’ (Group Interview 6). One participant assumed that the psychiatrist shown in our research materials had ‘seen a lot of different cases of mental health’, noting that mental health issues ‘can happen to anyone’ (Group Interview 4).

Factual knowledge, such as the description of symptoms, was considered helpful for those seeking to understand their own behaviour or that of others. As one person put it, the information about symptoms would ‘help me understand what was going on’ (Individual Interview 3) However, offering factual information on its own was considered potentially harmful. One young person described videos listing ‘signs’ of mental health conditions such as bipolar disorders as ‘leaving things on a cliff hanger’. They considered this a form of ‘baiting’ that might leave the audience ‘in a hopeless place that can be difficult for them.’ (Group Interview 13).

The young people reported a range of evaluative actions^44^ that they would use to assess credibility and reliability of the influencers as sources of information, including external cross-referencing (LinkedIn, NHS), cross-platform profile consistency checks, and monitoring internal metrics like follower counts ‘if there are lots of people following someone [..] it’s fair enough’ (Individual interview 6) and comment sections, ‘I’d check all the comments and see what people are saying, what their opinions are’ (Group Interview 9). Some young people considered visual cues and performance as evidence of reliability, such as a ‘professional looking bio’ (Group interview 5), being dressed in an ‘NHS uniform’ (Group Interview 3) or simply presenting with confidence, as opposed to ‘reading off a script’ (Group Interview 12). However, while visual content influenced the likelihood of them engaging with content, it was noted as problematic that ‘some people just look at the aesthetics and don’t even question what the influencer is saying.’ (Group interview 14).

In contrast to reliability, relatability was a quality associated with experiential information. Perceived relatability determined whether the young people wished to engage with mental health content: ‘In my opinion, if someone relates to a topic, they’re more likely to listen and understand it.’ (Group Interview 17) The perceived relatability of the influencer also increased the potential for social trust from young people. One participant from Group Interview 13 evaluated the lived experience and wellness influencers as more relatable, explaining, because ‘I feel like I have more in common with them, I feel like I can trust them more’ (Group Interview 13). Relatable stories of personal experience with mental health issues were valued as a template for self-help. When talking about a ‘day in the life’ example of a recovery story, one person described video as ‘step by step’ model for others, showing ‘this is the way I deal with anxiety, and this is how you could possibly do so too.’ (Group Interview 5) Practical, achievable examples of the creator demonstrating how to cope with mental health issues were widely considered more effective than professional advice, illustrated by the following participant’s opinion: ‘More people are inclined to not listen to educational videos but something that would relate more to their daily life.’ (Group Interview 12).

However, young people also acknowledged the limits of relatability, noting that personal experience is not necessarily generalisable. As one participant observed, influencers are ‘talking about their experiences, which aren’t the same as other people’s.’ (Group Interview 7) This was linked to the individualised nature of mental health, with another participant explaining that ‘certain methods of dealing with mental health might work for some people but then it doesn’t for others.’ (Group Interview 2) Relatability based on the shared characteristics of the influencer and audience was by no means universal. The limited range of mental health influencers that young people had encountered was considered problematic. Individual Interviewee 3 described the typical characteristics of mental health influencers as ‘usually people who are younger and usually female, and it’s usually that kind of demographic, and it would be good to get more diversity in who’s actually speaking about it.’ Key gaps in relatable content were found for young men, whose engagement with mental health content led to them ‘being hated on’ (Group Interview 17). The aspirational lifestyle solutions to mental health challenges narrated by influencers were also critiqued as ‘unrealistic’ and ‘unachievable’ for ‘people from disadvantaged backgrounds.’ (Group Interview 14).

Relatability was understood in terms ‘genuine’ disclosures of personal experience (Group Interview 18). In contrast to the cues of reliability, the visual cues of authenticity included evidence of spontaneity and affective displays e.g. ‘talking a lot more from emotion’ (Group Interview 12). However, this perception of authenticity was couched in wider distrust of a selective, ‘very subjective’ output created as attention-grabbing ‘theatrics’ to ‘get people talking about them’ (Group Interview 5), recognising that ‘with a camera you can capture what you want to and what you don’t’ (Group Interview 12). One participant noted that ‘you can’t trust whatever you see on social media, because sometimes they might be saying something, but that’s not actually what’s happening. They might be doing it for the benefit it brings them.’ (Group Interview 16) Despite this, selectivity was not always a matter that required moral evaluation as deceptive or misleading. Some young people felt that ‘playing it for the camera’ was laudable since they were ‘only there for the drama’ (Group Interview 17).

### 3.2. Theme 2. Beliefs about platforms and influencers: ‘TikTok’s fun but fake’

The critical thinking that young people brought to their evaluation of mental health information from influencers was shaped contextually by their dispositions towards platforms and their expectations of what they considered different sites to be ‘good for’. Young people described TikTok as ‘a very unserious place’ (Group Interview 13) used for fun and ‘joking around’ (Group Interview 5), which they accessed during their downtime, such as ‘on the way to school and back’ (Group Interview 13) or ‘before I go to bed’ (Group Interview 17). As a source of casual entertainment, TikTok was used for pleasurable distraction, whether that was in the form of ‘comfort’ (Group Interview 1) or escapism into ‘brain rot’ (Group Interview 14). This is in line with the platform vernacular of TikTok^40^ being strongly associated with entertainment.^55,56^

There was widespread consensus that TikTok was not a site to become better informed through formal learning activities. As one participant put it,

I just don’t go on TikTok expecting to learn information about mental health and I don’t really know who goes onto TikTok purely for educational purposes (Group Interview 16).

Instead, ‘educational’ use of online health content was associated with intentional, long-form viewing practices on YouTube, which they regarded as better suited to helping young people ‘process things’ (Interview Participant 3) and ‘understand [content] better’ (Group Interview 16). Notably, mental health content was regarded as a source of concern, at odds with the playful nature of TikTok. As one young person from Group Interview 5 said,

[on TikTok] you wouldn’t search for anything serious. Like you wouldn’t be searching for anything you’re genuinely worried about. It would be like oh let me find this meme or let me find this joke or something like that.

Instead, in line with previous research^57^ searching for medical information was associated with search engines like Google. One young person compared the light-hearted nature of TikTok with external search engines, which were for ‘more serious things… I just think Google’s the better one to be honest.’ (Group Interview 5).

Beliefs about influencers went hand-in-hand with the platform imaginary of TikTok. Influencers were primarily seen as figures associated with leisure pursuits who young people watched based on their appeal as funny, inspirational or sources of recommendation about niche interests such as fashion and lifestyle content, sports and gaming, food and fitness. Very few of the young people in our dataset were familiar with mental health influencers, although they mentioned certain professionals as recognisable psychologists or doctors who gained prominence in the Covid-19 Pandemic. The follower size of influencers also affected their appeal and perceived authority, though not always beneficially so. Some young people tended to follow micro-influencers, rather than celebrities or macro-influencers, explaining, ‘I don’t see the value in following people with loads and loads of follows. I feel like often they’re producing content that’s unrealistic,’ or ‘doing it for like, money’ (Group Interview 14). This was expressed more intensely in their criticism of commercialized mental health content, seen as exploitative ‘advertising’ (Group Interview 7), ‘doing it for the views’ (Group Interview 12) or as ‘marketing tactics’ (Group Interview 15). In this context, high numbers of followers rendered influencers ‘out of touch’, which one young person noted ‘just feels very fake to me, like just for the money and for fame.’ (Interview Participant 3).

### 3.3. Theme 3. Platform designs are tricky

A core element of TikTok is its algorithmically driven recommendation system, often referred to by young people simply as ‘the algorithm’, which they related to the content they saw on their ‘for you page’. The young people were adept at using functional skills to select, curate and strategically disengage from the mixed content that was pushed to them via the algorithm. Their knowledge of these skills was related to their algorithmic literacy,^32^ especially how they might select content to ‘train’ the algorithm and personalise their viewing feed. One participant from Group Interview 12 described this process:

The algorithm kind of brings them up. Like if you interact with videos, say with mental health, you will suddenly just get loads of videos in your stream about mental health as well.

On one hand, the algorithm was viewed positively as a way of helping people find content they are interested in. Some personalised the algorithm as ‘listening’ to them, which they found ‘kind of reassuring, because it’s like, you’re not alone’ (Group Interview 18). But others regarded it as ‘treacherous’ and ‘just awful’ (Group Interview 12). In part, this attitude was based on a perceived lack of moderation resulting in access to age-inappropriate content. More generally, the ever-changing nature of the content pushed to the ‘for you page’ meant the ‘fluctuating recommendation relevance’ associated with TikTok’s algorithm^44^ reduced young people’s critical evaluation of mental health content. As one participant put it:

Like even if you search something it will come up more on your for you page but you could go one minute watching a video about anxiety, two scrolls later you’re watching like some random like TikTok, just a joke or something. So like you never actually take it seriously I don’t think (Group Interview 10)

The process of tailoring the content in their ‘for you page’ presented an ongoing struggle to the young people in our data. The functional skills they used to ‘train’ the algorithm (Group Interview 15) entailed a range of socio-technical ‘friction’.^58,59^ Practices such as scrolling past to disregard content was a relatively frictionless practice that led to increasing speed of consuming content, such as ‘fast scrolling’, watching videos on double speed, or skipping parts of a video. In turn, this was a barrier to critical thinking. Viewing fast-moving streams of content led to ‘a lot of impulsiveness, rather than having to weight it all.’ (Group Interview 5).

In contrast, using the ‘not interested’ button, a function with the explicit purpose of deselecting specific videos or creators requires conscious effort on the part of the viewer, who needed to ‘then scroll back up just to press not interested’ (Group Interview 13). For some young people, the delays caused by this point of friction meant they were further exposed to unwanted mental health content due to TikTok’s default sound setting being ‘on’. Within the individual interviews, these risks were explored in more depth. For example, Interview participant 13 explained that when trying to avoid eating disorder content they experienced distress while hearing the voice-over playing by default,

because it takes a few seconds to, you know, get up the screen, you’re still hearing like, ‘so I weigh this many kilos’ and then it’s like, shut up. Oh my God.

The practice required to adjust sound settings and so avoid this risk was yet higher in terms of friction, requiring six steps from the point of opening the app to selecting the option within the playback options to ‘open TikTok on mute’.

Another practice used by the young people to avoid content that might harm their mental health was strategic disengagement from the app. At the time of data collection, there were in-app options to restrict viewing, through ‘scheduling a break’, a function located within TikTok’s settings. None of the young people in our data seemed aware of this function, though many argued that tech companies should provide similar interventions such as pop-up warnings when a phone was being used over a certain time. Others described external support from professionals that prompted their strategic disengagement from the app. Interview participant 13 explained, ‘My therapist told me kind of get off your phone and go do something else.’ This suggests gaps in young people’s media literacy relating to the design features of specific apps and how they might be used to improve mental health and wellbeing.

Functional skills for selecting content included curating content by marking it as a ‘favourite’. These gave young people some agency in personalizing their feed. However, searching and saving content might be motivated by a range of factors, not all of which were positive. In some cases, participants spoke about curating eating disorder content, to increase the algorithmically driven content they would then view to ‘punish’ themselves (Interview Participant 6). This suggests that knowing *how* to use the affordances of a site to save mental health information is not enough: young people also need to understand the motivations for their practices and the (dis)benefits this might bring. This requires a different aspect of critical thinking, informed by socio-emotional intelligence and tailored to the individual ways in which content about mental health can be harmful in certain contexts.

## 4. Discussion

### 4.1. Principal findings

This study is among the first to explore the ways in which young people’s perceptions of mental health influencers take place in the context of specific social media platforms. Our findings reveal a striking contrast between the platform beliefs and constraints of TikTok and its potential as a site for critical engagement with mental health content. In answer to RQ2, our participants overwhelmingly saw the site as a place for ‘fun’, ‘drama’ and ‘non-serious distraction’. This platform imaginary^39^ is at odds with the intentional attention required for formal health support, which our participants suggested they would seek in spaces generally dedicated to longer-form content. Secondly, the platform affordances of TikTok encourage passive consumption within its noisy algorithmic feeds that hinder critical thinking. Although young people were confident in their functional skills to search and select content, there were seeming gaps in their knowledge about the features within the app that might help them manage their mental health and wellbeing, such as setting time limits, muting the sound and refreshing their feed rather than using the ‘not interested’ button to constantly monitor and ‘train’ the algorithm away from content they considered unhelpful for their mental health. The algorithmic literacies of young people need further development to critique the effects of the apps that they use, as well as the content that they consider.

In answer to RQ 1, our results highlight an important literacy-action disconnect in the skills young people brought to evaluating mental health content. While the young people demonstrated a sophisticated vocabulary used to evaluate mental health content in terms of its reliability, this knowledge did not always translate into the skills needed to verify the credentials of content creators. Some participants assumed they could verify professional credentials using external sources, such as the NHS website. However, in the United Kingdom, health professionals are not listed on that site but instead on registers, such as those maintained by the General Medical Council. Nor does this account for professions that rely on voluntary registration with non-medical organisations (e.g. psychotherapists), or for regulatory practices that differ across national contexts. Instead, young people relied on information available within the platform, such as comment threads or the size of the influencer’s follower list. Such information was interpreted variously by young people, making influencers ‘unrealistic’ for some, while functioning as social proof for others. The basis for assessing relatability was also variable. Because relatability depends on the perceived similarity between the influencer and a young person, it is inevitably limited by the uneven representation of certain groups within the mental influencer landscape, and more generally by the subjective ways in which an individual will experience mental health.

### 4.2. Comparisons to other research

Academic research about influencers as conduits for health communication has burgeoned in recent years. Most of this has focused on influencer content^17,45^ or the perspectives of influencers themselves.^25,26^ Qualitative research about youth perceptions of mental health content from influencers is in its infancy.^26^ This study is the first to focus on the role that platform beliefs and design play in the way young people respond to mental health influencers, comparing content from health professionals, wellness practitioners and lived experience creators. Given that young people do not experience social media homogeneously, taking a platform-sensitive approach to this topic is vital, especially because the design and imaginary of sites like TikTok run counter to using the site for seeking reliable mental health support. This sounds a salutary note of caution to emerging claims that valorise influencers as a new paradigm for health communication,^15^ indicating that if we are to ‘meet young people where they are’^4^ we need to do so with their platform beliefs and practices in mind.

Our results support earlier suggestions that influencers can be of some value as sources of mental health information,^10^ particularly when they are considered relatable peers.^11^ However, while professionals may attempt relatability,^12^ our findings also show that professionals are valued more for their expertise and objectivity rather than shared experience. Importantly, participants both praised relatability and found it problematic. The affectively driven, interpersonal criteria used to assess relatability, coupled with a willingness to accept ‘drama’ as part of the entertainment-seeking uses of TikTok suggest that relatability within health communication needs a more critical lens than taken previously.

In line with research about misinformation about mental health,^48^ youth expressed concerns with mental health content that lacked reliable evidence and some scepticism about whether the credentials of health professionals could be trusted online. However, their confidence in how to check such credentials was somewhat misplaced, leaving them to rely on variable measures such as comments and follower counts to assess trust. Like other researchers,^44^ we argue that platform level interventions, such as robust verification systems and recognisable quality marks^60^ are much needed to support the media literacy of the people using the site for mental health information and increase trust in health professionals communicating online.^61^

### 4.3. Strengths and limitations

This is a robust qualitative study, with a large number of participants, that ensured a diverse range of perspectives that were enabled through group and individual options for participation. It draws on our previous, extensive mapping of influencer communication in TikTok^49^ and the active involvement of a diverse YPAG to support data quality.

While this research has offered important contributions to our understanding of how mental health communication from influencers is perceived by young people, there are inevitable limitations to this study. Our decision to anonymise data at the point of transcription meant we could not return transcripts to all participants for checking, meaning we may have missed some contextual feedback helpful to further improve data quality. Our data focused only on the use of TikTok, English language posts, and a population of 16 to 25-year-olds in the United Kingdom. The digital landscape changes rapidly, and the rise of LLMs and artificial intelligence systems further complicate the ways influencers might create content and be perceived by their audiences. Future research should go on to explore the contextual variation that impacts the potential for mental health literacy across other age groups, neurodiverse groups and investigate the perceptions of those with lived experience of specific mental health issues. We also call for research investigating mental health influencers who use different languages and platforms and operate in different national contexts so that we might maximize the potential for effective mental health communication on a global level.

## 5. Conclusion

Our study has provided the first, empirical account of the platform-sensitive ways young people in the United Kingdom respond when they encounter mental health content from social media influencers on TikTok. The data show both a nuanced picture of the media literacy skills that young people employ when navigating influencer-driven mental health content and, importantly, the beliefs that inform what content young people seek to engage with on TikTok and the role this plays when they encounter health information.

Our findings present two novel contributions. First, in response to RQ1, we show that young people possess a sophisticated understanding of misinformation and are familiar with key verification practices, such as factchecking. The young people tended to articulate a vocabulary of critical evaluation, including searching for common indicators of trustworthiness and recognising the importance of assessing source expertise. However, this knowledge did not always translate into actionable strategies when applied to the context of influencer culture broadly and TikTok’s platform affordances in particular. This disconnect was further exacerbated by the points of friction presented by TikTok. The young people voiced an intimate familiarity with ‘the algorithm’, TikTok’s algorithmically driven recommendation system, but expressed their continuous struggles trying to ensure that the content they saw adhered to their interests and to disengage when faced with harmful content.

Second, in response to RQ2, we highlight the challenge of platform beliefs and affordances on the perceived use of TikTok as a delivery channel for health information. While the young people viewed influencer-led mental health content through a lens of reliability and relatability, this was preceded by a platform-level belief that TikTok is meant for light entertainment rather than education. This view of TikTok is in line with platform affordances of TikTok, which further hinder engagement with and critical evaluation of educational health communication, with its short form posts, noisy flows of algorithmically driven content and socio-technical frictions that encourage passive consumption. To that end, we believe that mental health professionals and public health campaigns need to take a more platform-sensitive approach to online communication, keeping in mind how the platform-driven cultural norms and beliefs shape the ways young people engage with content.

## Supplemental material

Supplemental material - Media literacies, platform imaginaries and mental health influencers: How young people respond to mental health content on TikTok Supplemental material for Media literacies, platform imaginaries and mental health influencers: How young people respond to mental health content on TikTok by Alex Christiansen, Ruth Page, Shioma-Lei Craythorne, Michael Larkin, Paul Crawford, Evangelina Asiedu-Addo, and Emma Garavini in DIGITAL HEALTH.
