# Supplementary material for: Media literacies, platform imaginaries and mental health influencers: How young people respond to mental health content on TikTok
Source: Digit Health. 2026 Aug 21;12:20552076261479043. doi: 10.1177/20552076261479043 (PMC13617548; doi:10.1177/20552076261479043)
Supplement: Supplementary file 1 [file sj-pdf-1-dhj-10.1177_20552076261479043.pdf]

Appendix

Appendix A: Topic Guide for Group and Individual Interviews

1. Could you tell us about some influencers you have heard about or seen?

Possible prompts: How did you come across them? What were your immediate thoughts about them? Why did you feel that way about them?

2. Do you recognise these influencers who talk about mental health? [Participants were shown a sheet with five illustrative examples of influencers].

Possible prompts: Where have you seen them before? What makes them recognisable to you? Would you recognise them in a different place (e.g. on an advertisement or poster?)

3. We will now show you some examples of posts by influencers who talk about anxiety/stress

[Trigger warning: young people were told that they did not have to watch the videos if this topic or the video content made them feel uncomfortable].

As a reminder, here are three cards to remind you of the different videos. We would like you to place them in order of how useful they are in terms of:

(1) Providing information about mental health

(2) Providing helpful information about where you could find help/support for mental health

(3) Changing attitudes towards mental health

[The proposed videos shown]

- Dr Jaleel Mohammed, talking about 'Five signs of Social Anxiety Disorder' https://www.tiktok.com/@drjaleelmohammed/video/7342249680765504801?q=dr%20jaleel%20mohammed&t=1727883624788
- Chef Bai, talking about chia bowls as an antidote to anxiety https://www.tiktok.com/@chef_bai/video/7241407563051470126
- Ro Mitchell talking about her daily experiences of anxiety.
- https://www.tiktok.com/@romitchell/video/7387451307306618145?is_from_webapp=1&sender_device=pc&web_id=7381487763836028448

[Follow up prompt] What made you come to those decisions?

4. [For each separate video] Who do you think the intended audience is for this video?

Possible prompts: What made you come to that conclusion? What do you think about the language they use? Why do you think this would or would not be aimed at [an older person, for example]? Have you seen videos like this before?

5. How would you interact with this video if you saw it online?

Possible prompts: Would you scroll past it? Would you save it to watch later? Would you send it to a friend? Or anything else? How did you feel when you watched this?

6. How do you think influencers might best help young people as resource for mental health: Which is most important - providing information, solutions or changing attitudes?

Possible prompts: Have you seen any videos by influencers that you thought were particularly helpful or unhelpful? What did they do or say in those videos that makes you think that? Are there any topics about mental health that you think influencers should talk about?

7. We'll be using the discussions today to help us prepare reports to go to policy makers. What would you most like policy makers to know about how mental health communication from influencers is shared in social media?

Do you have any questions for us?

Appendix B: Code book

GI = Group Interview

II = Individual Interview

Theme 1. Reliability and relatability are criteria for assessing mental health content.

Subtheme 1.1 Reliability as a quality used to assess mental health information

1. Factual information is more or less reliable

   I think this video is like, it's more factual. So it's probably like, more reliable. (GI 6)

2. Factual evidence is verifiable

   well I guess he doesn't really have evidence or proof but it kind of – she was just saying, this breakfast will make you feel less anxious. (GI 10)

3. Unreliable information is harmful

if you don't have the proper research and then someone suffers because of your misinformation, I feel like that's a really big thing. (GI 16)

4. Reliable information is useful for understanding mental health conditions

   If I was struggling with that, it would actually help me understand what was going on (II 3)

5. Information on its own is not enough

   this [video] is more informational, but when you're struggling in that thing, I don't think information can really help you that much. (GI 16)

6. Professional qualifications increase the creator's reliability

   he just has the qualifications, he knows what he's talking about, so it just, you know, seems more professional and easily believable (GI 15)

Qualifications can be assessed for their accuracy

7. From recognised authorities outside social media

   But I would personally go to the NHS website anyway, then I'd be like, 'Okay, cool, they were telling the truth.' (GI 12)

8. Cross-referenced within social media content

   You look at many apps and see if this person is credible (GI 2)

Drawing on the 'wisdom of the crowds'

9. Followers

   I would say check their followers to see if they're credible (GI 6)

10. More followers indicate more credibility

    So if there are lots of people following someone, and I think the person's content is, well, it's fair enough. (II 6)

11. Comments

    Actually, I'd check all the comments and see what people are saying, what their opinions are and whether it actually does have an effect. (GI 14)

12. Reliability from professionals is distinct from personal experience

    And it was like a doctor, it wasn't someone with anxiety, so it was like an outside perspective. (GI 6)

13. Professionals can be trusted because they have treated many people

    The doctor might have a bit more of a generalized view because he's seen a lot of different patients who suffer from X, Y, and Z in terms of mental health. So, he could probably adequately help you, (GA 16)

14. Professionals are helpful for a general audience

    so the doctor is for everyone (GI 7)

15. Mental health issues can happen to anyone

    he's making you aware of anxiety which can happen to anyone. (GI 4)

Reliability can be assessed visually by

16. Profiles are used as a source for credibility

    *I just go into their profile and see like if they've got this like professional looking bio**.** (GI 9)*

17. The more confident the delivery the more reliable the creator

    he's reading off a script, you can literally see his eyes look back to what he's doing (GI 12)

18. Setting and dress imply credibility

    I follow one doctor and he's always creating videos while he's working. He always has his NHS uniform on and that's one of the ways I'd identify someone. (GI 3)

19. Visual cues can be misleading

    some people just look at the aesthetics and don't even question what the influencer is saying. (GI 14)

20. Online credentials are not always reliable

    I can definitely see how sometimes, like, you know, being a doctor doesn't really mean that.(II 12)

Subtheme 1.2: Relatability is a quality that is used to assess mental health content

21. Relatability is based on shared experience

    I feel like it's more important to make young people feel relatable. And they feel like they find someone on social media that has the similar experience to them. (II 6)

22. Relatability presumes authenticity

She's willingly posting this, she genuinely has anxiety and it's her experience (GI 2)

23. Authenticity is shown through emotional spontaneity

    She's talking a lot more from emotion, you can tell it's a lot more from emotion, than the doctor just going, 'This is how you can tell social anxiety.' (GI 12)

Relatability has positive outcomes

24. Relatability increases trust, especially at an emotional/social level

    But, like, these two women, especially because I feel like I have more in common with them, I feel like I can trust them more, like, on a social level, rather than a doctor. (GI 8)

25. Relatable content allows you to take practical steps in your own life

    you might find it relatable, because it's something you can do too. (GI 6)

Relatability is limited

26. Personal experience is always documented in a selective manner

    like with those kind of videos, you don't know if that's actually like their life, because sometimes they're playing it over the camera (GI 12)

27. Relatability is not representative of all social media users or all problems

    she only speaks about one problem that she had one time and that's just completely unrelatable. (GI 17)

28. People's experience of mental health can vary

    with anxiety, it can differ from person to person how they deal with it (GI 3)

29. Relatability can be limited by access to resources,

    in terms of the chef, like, I don't own, I don't have chia seeds or dragon fruit or any of that at home. (GI 5)

Theme 2. Platform beliefs shape the evaluation of mental health content

30. You cannot trust anything you see online

    I think you can't always trust what you see on social media anyway. (GI 12)

31. TikTok is fake

the reason I don't really use Tiktok is because it all just feels very fake to me, and it feels very like. Just for money and and just for fame (II 3)

32. 'Doing it for the views' is a source of distrust

    It just felt like she was doing this for views, she didn't actually care about the meaning that other people would be watching her video for. It just felt like this is an easy way to get clicks, get views and all that. (FG 17)

33. TikTok is for entertainment

    when I do TikTok I find – I look for entertainment (FG 1)

34. TikTok is for downtime

    I only watch TikTok sometimes when I just want to while away time, pass time, (FG 12)

35. TikTok is not for education

    No, I just don't go on TikTok expecting to learn information about mental health and I don't really know who goes onto TikTok purely for educational purposes. (FG 16)

36. Google is better than TikTok for education

    TikTok best for like joking around I wouldn't say it's not a serious app compared to Google where because with Google you can actually have proper studies put on. (*FG 6)*

37. Influencers are associated with leisure interests

    I usually follow like fashion and like lifestyle stuff. So it doesn't really matter to me as long as I find the person funny. (FG 14)

38. The greater the visibility/follower count, the less realistic the influencer

    because I don't really see like the value in following people with like loads and loads of follows. Just because I feel like often they're producing content that's like unrealistic (FG 13)

Theme 3. Platform designs impact engagement with mental health content

Beliefs about the algorithm

39. Algorithms push a stream of content to you.

Yeah, the algorithm kind of brings them up. Like if you interact with videos, say with mental health, you will suddenly just get loads of videos in your stream about mental health as well. (FG 18)

40. Training the algorithm requires skill

    these women, who both seem slightly younger, and know how to use the TikTok algorithm (FG 15)

41. Training the algorithm is ongoing and takes effort

    So I had to like retrain my whole phones like not give me this stuff instead of me. (II 13)

42. Training the algorithm can be motivated negatively

    I would like copy the link and stuff […] then it would feed me more because like it's like what I was saying like I want to to punish myself (II 4)

43. The algorithm can be shaped by social practices

    I think on TikTok, as well, when somebody reposts something, and, like, say I like it, it automatically makes us share the same algorithm. (FG 12)

44. A personalised algorithm can be positive

    I always like say to my friends, I was like, they're listening to me. But like, at the same time, it's kind of reassuring, because it's like, you're not alone. (FG 18)

45. The algorithm is dangerous

    Their algorithms are treacherous. (FG 12)

46. Algorithmic flows of information are very mixed, which prevents serious use

    Like even if you search something it will come up more on your full page but you could go one minute watching a [30:11] about anxiety, two scrolls later you're watching like some random like TikTok, just a joke or something. So like you never actually take it seriously I don't think. (FG 5)

47. Scrolling past is a passive strategy to indicate disinterest

    I wouldn't watch it, I'd just scroll past.(GF 2)

48. Marking content as 'not interested' takes effort

Yeah. I'd read five signs of social anxiety disorder, scroll past and then scroll back up just to press 'not interested'. (GI 13)

49. The 'default sound' is dangerous

But then like because it takes a few seconds to, you know, get up the screen, you're still hearing like, so I weigh this many kilos and then it's like, shut up. Oh my God. (II 13)

50. Choosing to disengage from devices and platforms for wellbeing requires intervention

[my therapist told me to] kind of get off your phone and go do something else. Which kind of worked quite well because it was like instead of then falling into this little trap of be like, Oh no, I'm going to look at this for three hours. I'd get up actually get up and do something. (II 13)
